# Regulating Tissue Growth Factors for Healing With Etherified Carboxymethylcellulose Matrix

**DOI:** 10.1093/jbcr/irae124

**Published:** 2024-07-02

**Authors:** Guiting Lin, Shandilya Ramdas, Hosam Hadid, Jared Van Vleet, Tom F Lue, Stathis Poulakidas

**Affiliations:** Knuppe Molecular Urology Laboratory, Department of Urology, School of Medicine, University of California, San Francisco, CA 94143, USA; Department of Surgery, University of Illinois Chicago, Peoria, IL 61605, USA; Department of Surgery, Arkansas College of Osteopathic Medicine, Fort Smith, AR 72916, USA; Office of Medical Student Research, Oklahoma State University Center for Health Sciences, 2nd Lieutenant, US Air Force, OMS-3, Oklahoma State University College of Osteopathic Medicine, Tulsa, OK 74107, USA; Knuppe Molecular Urology Laboratory, Department of Urology, School of Medicine, University of California, San Francisco, CA 94143, USA; Burn/Wound Care Services, Stroger Hospital of Cook County, OSF/St. Anthony’s Medical Center, Rush University, Chicago, IL 60612, USA

**Keywords:** etherified carboxymethylcellulose matrix (eCMC), cytokines, regeneration, burn model, mechanism

## Abstract

Etherified Carboxymethylcellulose Matrix (eCMC) is a revolutionary application of carboxymethylcellulose (CMC) in wound care, known for its potential in hemostasis and tissue regeneration. This study aims to investigate the mechanism of eCMC in tissue healing by establishing a rat burn model and administering eCMC as a treatment. The objective is to analyze cytokines and inflammatory mediators using a Cytokine Array and histochemical staining to understand the effects of eCMC on tissue regeneration. A rat burn model was created, and eCMC was applied as a treatment. Tissue samples were collected at multiple time points to assess the expression of cytokines and inflammatory mediators using a Cytokine Array. In addition, histochemical staining was performed to evaluate tissue regeneration factors. eCMC induced the expression of endogenous cytokines, particularly vascular epithelial growth factor and platelet-derived growth factor, while inhibiting inflammatory cytokines such as CINC-1, CINC-2, and MMP-8. This dual action facilitated wound healing and mitigated the risk of infection. eCMC demonstrates promising potential for enhancing skin regeneration. Further research is warranted to delve into the precise mechanism of eCMC’s cytokine regulation. In vitro and in vivo studies should be conducted to comprehensively investigate the therapeutic capabilities of eCMC in wound healing.

Etherified Carboxymethylcellulose Matrix (eCMC) represents a cutting-edge and highly innovative application of carboxymethylcellulose (CMC) in wound care, specifically for the purposes of hemostasis and tissue regeneration.^1–3^ Originally designed primarily for hemostasis, eCMC has demonstrated remarkable efficacy in various clinical scenarios, including renal hemostasis^4^ and the control of peripheral vascular bleeding.^5^ Extensive research has revealed that eCMC offers significant advantages in terms of its hemostatic properties, particularly when compared to other existing hemostatic products currently available on the market. According to recent preclinical and clinical studies, eCMC was widely used for prehospital hemorrhage control, for full-thickness burn wound debridement, major topical bleeding, donor site healing in burn graft harvests, deep partial-thickness burns, major bleeding in damage control surgery in abdominal trauma and chronic wounds.^6^ This synthetic polymer has been shown to be suitable for hemostatic application, wound sealing, and protection for skin wounds, such as burns, with studies in the animal model demonstrating healing cytokines and antiinflammatory mediators in the wounds.^7^

Previous research has extensively investigated the nanostructure of the Etherified Carboxymethylcellulose Matrix (eCMC). Encouraging preclinical findings have demonstrated the efficacy of eCMC in facilitating burn wound healing in two animal models.^6^ Subsequent clinical trials have provided further evidence supporting its effectiveness, as eCMC has exhibited the ability to reduce bleeding recurrence and alleviate burning/pain sensations following application. In addition, notable antiinfective effects have been observed. Even more strikingly, eCMC promotes skin epithelization and reduction of scar tissue in burn wounds.^6,8,9^ Recent observations have highlighted the remarkable potential of this polymer in stimulating adipose-derived stem cell proliferation and facilitating wound healing.^3,10^ Notably, it exhibits a unique binding affinity for the epithelium, promoting cell migration and facilitating tissue regeneration. Over the years, carboxymethylcellulose (CMC) has been used as a standalone material, with or without the incorporation of drugs and co-excipients, for the treatment of partial-thickness wounds, diabetic ulcers, foot lesions, and deep dermal filler applications. These beneficial effects have been observed at both donor and acceptor sites. In vitro investigations have further elucidated that eCMC promotes mitosis and proliferation of skin stem cells in a dose-dependent manner. Intriguingly, the product triggers activation of the Wnt/β-catenin signaling pathway through AMPK, potentially serving as the underlying mechanism by which eCMC stimulates the activation of stem cells and dermal cells, thereby promoting the process of wound healing.^10^

Hence, the remarkable potential of eCMC and its related products to stimulate dermal and stem cell proliferation makes them promising candidates for enhancing skin regeneration following skin injuries or burns. The activation of resident stem/progenitor cells represents a novel approach for skin stem cell therapy. However, achieving this task, particularly through the modulation of tissue growth factors, currently lacks a widely accepted method.

In current study, we established a rat burn model and administered eCMC as a treatment for the burn injury. Tissue samples were collected at various time points to conduct an analysis of tissue cytokines and inflammatory mediators using a Cytokine Array. In addition, histochemical staining was performed to identify factors associated with tissue regeneration.

## METHODS

### Materials and establishment of animal model of burn injury

The Etherified Carboxymethylcellulose Matrix (eCMC) eCMC in the form of BloodSTOP IX was provided by LifeScience PLUS, Inc., CA, USA. The Sprague-Dawley (SD) rats with 11 weeks old were bought from Beijing Huafu Kang Biological Technology Co., Ltd., Experimental Animal Production License Number: SCXK (Beijing) 2019-0008. All experimental procedures were adhered to, according to the National Research Council’s Guide for the Care and Use of Laboratory Animals and were approved by Scientific Investigation Board of Tianjin Yishengyuan Biotechnology Co., Ltd. (Experimental Animal Use License Number: SYXK (Tianjin) 2021-0003, Experimental Animal Qualification Certificate Number: 110322210103278136.Tianjing, China). Through the duration of the investigation, rats were fed in groups of 3–4 per cage, kept on a 12-h light/12-h darkness cycle, as well as had unrestricted access to food and water. A total 42 SD rats weighing 190 ± 10 g were randomly divided into 8 groups including sham group, control groups and experimental groups (time points 3, 14, and 20 days): (1) Control surgical group (N); (2) Control Vaseline gauze-1 (Vaseline-1): 3 days; (3) Control Vaseline gauze-2 (Vaseline-2): 14 days; (4) Control Vaseline gauze-3 (Vaseline-3): 20 days; (5) eCMC-1: 3 days; (6) eCMC-2: 14 days; and (7) eCMC-3: 20 days.

The rats were prohibited from water ingestion for 12 h before burns injury. The dorsal area of each rat was shaved, and a burn injury was induced by exposing it to 100 °C water in a flask for 8 s,^11^ allowing for the development of a deep partial-thickness/near full-thickness burn. Following the modeling process, the control Vaseline gauze group had the injured site covered with two layers of Vaseline gauze, the control regular gauze group had the injured site covered with two layers of regular gauze, and the experimental group was covered with two layers of eCMC. All wounds were then wrapped with gauze and securely fixed. Daily observations were conducted, and images were captured to monitor the progression of the injury. Beginning from the second day, the dressings were changed daily, and photographs were taken regularly to document and track the wound healing process (Supplementary Figure S1). Skin specimens from both the control and treatment groups were collected on days 3, 14, and 20 after treatment in the injury model.

To collect tissue samples from the rats, the animals were anesthetized using a 10% chloral hydrate solution administered via intraperitoneal injection. Tissue scissors were employed to carefully extract tissue specimens, approximately 5 mm away from the edges of the burn wound. The collected samples included the entire thickness of the skin, encompassing both the superficial layers and the underlying muscle tissue. These tissue samples were subsequently preserved in Bouin’s Fixation with liquid or Zenker’s solution for histological analysis. In addition, PBS was used as a preservation medium for the tissue samples intended for the Cytokine Array study.

### Histology and immunohistochemistry

The evaluation of various parameters was performed to assess the integrity of the skin structure, including the presence of neutrophils, fibroblasts, capillaries, and the repair of the dermis, epidermis, and skin appendages. In addition, bacterial presence within the tissue was also examined. The HE staining of tissue sections were performed.

For the immunohistochemistry, the paraffin sections were dewaxed following the antigen retrieval. It was important to prevent excessive evaporation of the buffer solution and ensure that the slides did not dry out during this process. Once naturally cooled, the slides were immersed in PBS (pH 7.4), shaken, and washed three times for 5 min each. Subsequently, the endogenous peroxidase activity was blocked. The primary antibodies, including PDGF (dilution ratio 1:50), EGF (dilution ratio 1:200), VEGF (dilution ratio 1:200), FGF-2 (dilution ratio 1:200), FGF-7 (dilution ratio 1:400), TGF-β1 (dilution ratio 1:500), and TGF-β3 (dilution ratio 1:200), were then incubated with the slides overnight at 4 °C in a wet box. Following incubation, the slices were washed three times with PBS (pH 7.4) for 5 min each. The corresponding secondary antibodies (HRP-labeled) for each species of primary antibody were applied to the slides and incubated at room temperature for 15 min. Subsequently, the horseradish enzyme-labeled streptavidin working solution was added and incubated at room temperature for 15 min to initiate DAB color development. Positive staining was indicated by a brownish-yellow color, and the sections were rinsed with tap water to halt color development.

For image analysis, five randomly selected fields per tissue were captured and recorded using a Retiga Q Image digital still camera and ACT-1 software (Nikon Instruments Inc., Melville, NY, USA). The differences in the expression of the 7 growth factors among the different groups of skin tissues were then detected and analyzed.

### Cytokine array

The total protein was isolated from tissues samples, each group of protein samples were pooled together, and cytokine array were used to detect cytokine changes between groups. The RayBio C-Series Rat Cytokine Antibody Array C2 (RayBiotech Life, Inc.Peachtree Corners, GA, USA) was used for the semi-quantitative detection of 34 rat cytokines, including Activin A, Agrin, B7-2, CD86, b-NGF, CINC-1, CINC-2 alpha, CINC-3, CNTF, Fas Ligand, Fractalkine, GM-CSF, ICAM-1, IFN-gamma, IL-1 alpha, IL-1 beta, IL-1 R6, IL-2, IL-4, IL-6, IL-10, IL-13, Leptin, LIX, L-Selectin, MCP-1, MIP-3 alpha, MMP-8, PDGF-AA, Prolactin R, RAGE, Thymus, Chemokine-1, TIMP-1, TNF alpha, and VEGF-A. The membrane of cytokine array was incubated with 2 ml of blocking buffer at room temperature for 1 h. After removing the blocking solution with a suction pump, add 1000 µl sample (the sample is loaded at 500 µg/ml). Cover with a lid, wrap with plastic film, and incubate at 4 °C overnight with shaking. After washing, the cytokine array was incubated with biotin-labeled antibodies at room temperature for 2 h. Then, 2 ml of HRP-streptavidin was added to each membrane and incubate with shaking at room temperature for 2 h. The color was developed with solution C/D mixture. Image Quant LAS 4000 scanning chemiluminescence imaging analysis system (GE Healthcare Corporate, USA) was used to capture the images. The analysis software that comes with the instrument is used to extract data, and the data analysis software of AAR-CYT-2 was used for data preanalysis.

For the differential gene expression screening, after the original data were normalized by software, select Normalization 2 without background data for analysis. Use fold change (fold change in expression) to screen differential proteins, and the selection conditions are as follows: (1) fold change ≤ 0.83 or fold change ≥ 1.2. (2) It was recommended to select an average (fluorescence) signal value > 150 per group. The scatter plot function is *ggplot2*, and the data package is *ggfortify*, which comes from R software.

Gene Ontology (GO for short) is an international standard classification system for gene function. GO can be divided into 3 parts: molecular function, biological process, and cellular component. Through GO enrichment analysis, it is possible to find important functions that lead to trait changes and find the genes corresponding to the functions. For the GO enrichment analysis of differential genes, the method adopted is Fisher’s exact test, and the data package is clusterProfiler, which comes from R/Bioconductor. The selection criteria are the number of proteins that fall on a certain term/GO difference ≥ 2, *P* < .05, The term/GO obtained in the drawing is sorted in descending order according to the value of Count, and the first 10 results are taken.

### Statistical analysis

Data were analyzed with Prism 5 (GraphPad Software, San Diego, CA, USA) and presented as means ± SD. Statistical significance between 2 groups was analyzed by the Student’s *t*-test. For statistical significance among multiple groups, one-way ANOVA analysis followed by Bonferroni post hoc analysis was performed.

## RESULTS

### The eCMC promoted tissue healing and angiogenesis

**Figure 1.**
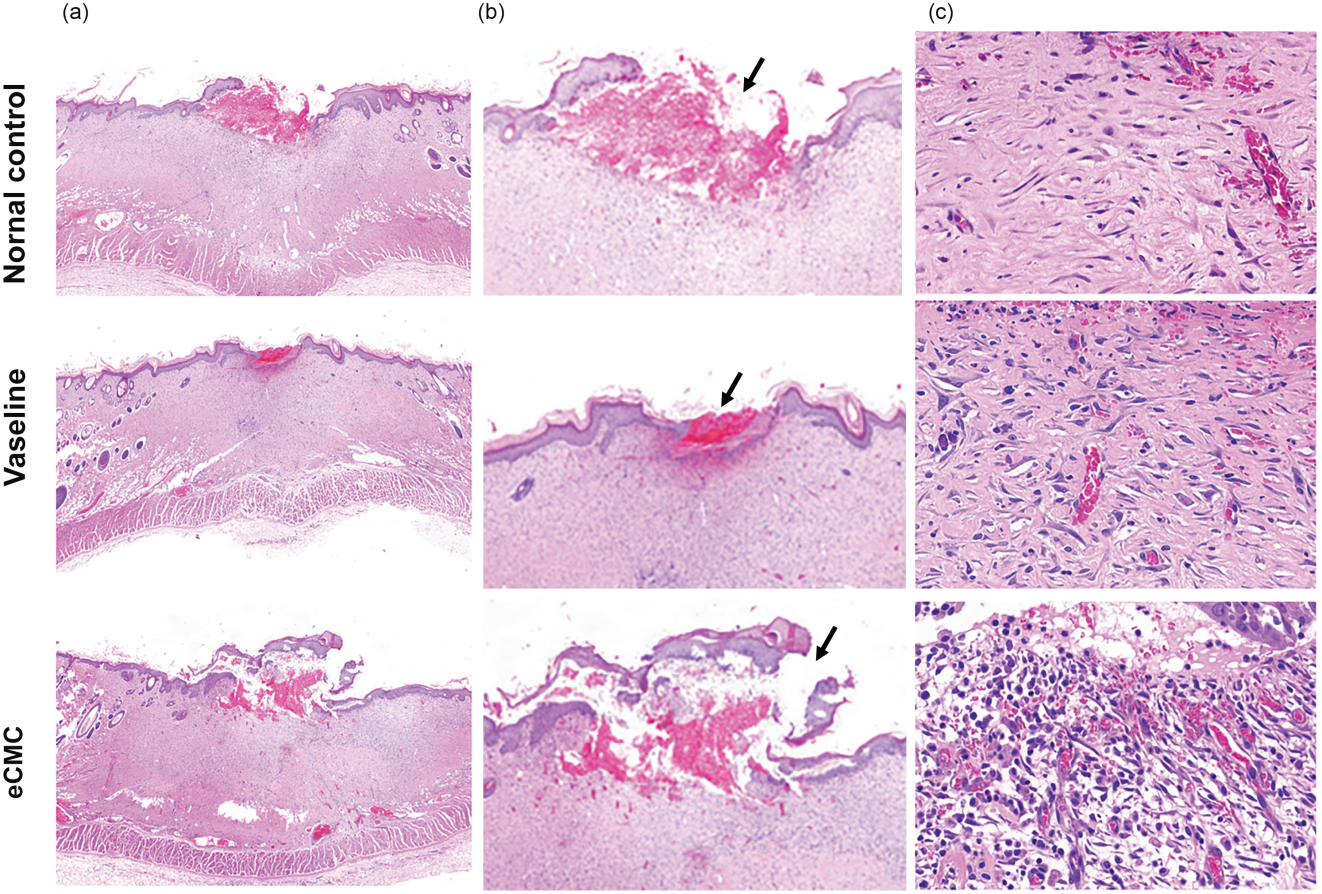
eCMC Significantly Improved Skin Healing Ability After Burn Injury. (A) Upon Closer Examination, (B) High-Magnification Images Revealed the Presence of Nascent Epithelial Cells That Overlaid the Granulation Tissue, Effectively Filling the Gap Between the Skin and Underlying Tissue (Arrow). (C) Moreover, eCMC Played a Significant Role in Promoting the Formation of Numerous New Blood Vessels Within the Subcutaneous Tissue, Further Supporting Tissue Regeneration

In this experiment, tissue paraffin sections were subjected to routine Hematoxylin and Eosin (HE) staining to evaluate tissue regeneration and vascular density in the subcutaneous tissue following a 20-day burn injury. The results revealed that, compared to the Vaseline gauze and sham groups, the application of eCMC significantly enhanced the skin’s healing capacity after burn injury, especially on the 7th and 14th days postexperiment, the eCMC burn model showed less localized inflammatory response around the burn wound area compared to other burn models. Notably, a newer epithelium with a crawling pattern was observed to migrate across the wound surface (Figure 1A and B). Upon closer examination at higher magnification, nascent epithelial cells were observed overlaying the granulation tissue, effectively filling the space between the skin and the underlying tissue.

Furthermore, eCMC exhibited a remarkable ability to promote the formation of numerous new blood vessels within the subcutaneous tissue, facilitating tissue regeneration and recovery following the burn injury (Figure 1C).

### The eCMC modulating tissue growth factors

**Figure 2.**
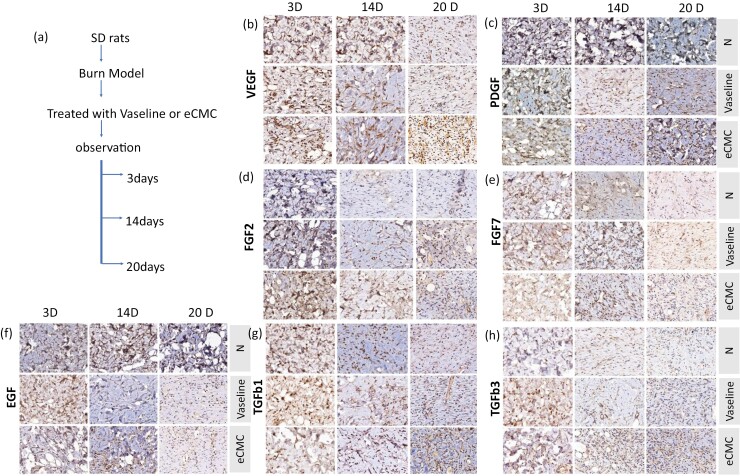
eCMC Increased the Expression of Endogenous Growth Factor in Subcutaneous Tissue. (A) Experiment Procedures. (B) Immunohistochemistry (IHC) Analysis of Vascular Endothelial Growth Factor (VEGF) Was Performed in the Normal (N), Vaseline, and ECMC Groups. (C) Expression of PDGF. (D) Expression of EGF. (E) Expression of Fibroblast Growth Factor (FGF2). (F) Expression of Fibroblast Growth Factor (FGF7). (G) Expression of Transforming Growth Factor β1 (TGF-β1). (H) Expression of Transforming Growth Factor β3 (TGF-β3)

**Figure 3.**
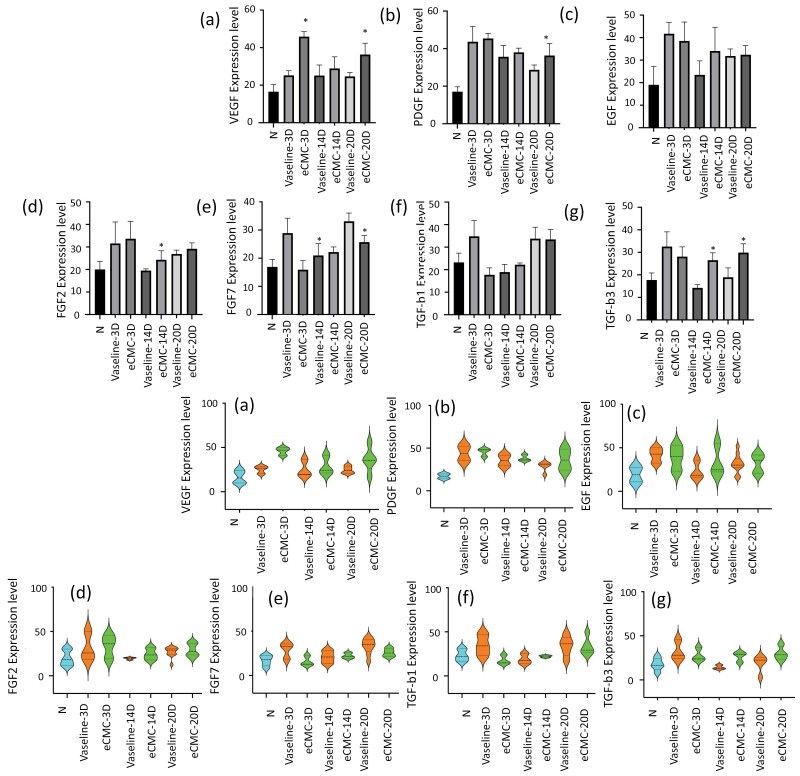
eCMC Increased the Expression of Endogenous Growth Factor (PDGF) in Subcutaneous Tissue. Up Panel Represented the Expression of Growth Factor in Bar Chart. Low Panel Represented the Data Distribution of Growth Factors in different Groups With Violin Plot. (A) The Expression of VEGF, Representing the Distribution of VEGF-Positive Expression Signals in Each Group (*n* = 6). The Y-Axis Represents the Percentage (%) of VEGF-Positive Expression Signal Value Relative to the Total Field of View (*P < .05). (B) The Expression of PDGF (*P < .05). (C) The Expression of EGF. (D) Expression of FGF2 (*P < .05). (E) Expression of FGF7 (*P < .05). (F) Expression of TGF-β1. (G) Expression of TGF-β3 (*P < .05)

In this experiment, the impact of eCMC on the expression of 7 endogenous cytokines in tissue was assessed by detecting these cytokines in tissue paraffin sections. As mentioned previously, 6 tissue specimens were sectioned from each group, and immunohistochemical (IHC) staining was performed (Figure 2). The stained tissue slides were then subjected to holographic scanning, and the resulting images were analyzed quantitatively to determine the extent of positive signals (Figure 3).

(1) *Effects of eCMC on vascular epithelial growth factor*

Vascular endothelial growth factor (VEGF) is a prominent growth factor that plays a crucial role in stimulating and promoting angiogenesis within the body. The results of the study demonstrated that eCMC had a significant impact on the expression of VEGF in the tissue compared to the application of Vaseline gauze. Specifically, on the 3rd and 20th days of application, eCMC markedly increased the expression of VEGF in the tissue (*P* < .01) when compared to the Vaseline gauze group. Furthermore, on the 14th day, eCMC continued to enhance the expression of VEGF in the tissue, although the differences were not statistically significant when compared to the Vaseline gauze group (*P* > .05). However, when compared to the normal control group, eCMC significantly increased the expression of VEGF in the tissue at 3, 14, and 20 days (*P* < .05) (Figure 3A). These findings highlight the ability of eCMC to positively influence the expression of VEGF, suggesting its potential role in promoting angiogenesis and supporting tissue healing and regeneration.

(2) *Effects of eCMC on platelet-derived growth factor*

Platelet-derived growth factor (PDGF) is a crucial growth factor involved in the regulation of cell growth and division. It plays a vital role in various processes such as angiogenesis (the formation of new blood vessels from existing ones), mitosis (cell proliferation) of mesenchymal cells including fibroblasts, osteoblasts, tenocytes, and vascular smooth muscle cells, as well as the chemotaxis and directional migration of these mesenchymal cells. The findings of the study indicated that eCMC exhibited an increasing trend in the expression of PDGF in the tissues on the 3rd, 14th, and 20th days of application. Although the differences did not reach statistical significance when compared to the Vaseline gauze group (*P* > .05), it is noteworthy that the effect was particularly prominent on the third day. In comparison to the normal control group, eCMC significantly enhanced the expression of PDGF in the tissues at 3, 14, and 20 days (*P* < .05) (Figure 3B).

These results suggest that eCMC has the potential to positively influence the expression of PDGF, indicating its potential role in promoting cell growth, angiogenesis, and the migration of mesenchymal cells.

(3) *Effects of eCMC on epidermal growth factor*

Epidermal growth factor (EGF) is a cytokine that plays a significant role in stimulating cell growth and differentiation through its receptor binding, and it is closely associated with tissue regeneration. The study results revealed that on the 14th day of application, eCMC demonstrated an increase in the expression of EGF compared to Vaseline gauze. Although it was higher than the expression observed with Vaseline gauze, the difference did not reach statistical significance (*P* > .05). On the 20th day, eCMC exhibited a slight elevation in the expression of EGF, slightly surpassing that of Vaseline gauze. It is important to note that on day 3, the effect of Vaseline gauze in promoting EGF expression appeared to be higher than that of eCMC, although the difference did not reach statistical significance (*P* > .05). When compared to the normal control group, both eCMC and Vaseline gauze increased the expression of EGF in the tissues at 3, 14, and 20 days, but the differences did not reach statistical significance (*P* > .05) (Figure 3C).

These findings indicate that both eCMC and Vaseline gauze have the potential to influence the expression of EGF in tissue, suggesting their involvement in tissue regeneration processes. However, further investigation is required to ascertain the significance and clinical implications of these observations.

(4) *The effect of eCMC on basic fibroblast growth factor*

Basic fibroblast growth factor (FGF2) is a cytokine with broad mitogenic and cell survival activities, playing a crucial role in various biological processes, such as embryonic development, cell growth, morphogenesis, tissue repair, tumor growth, and invasion. The study results indicate that eCMC led to an increase in the expression of basic fibroblast growth factor (FGF2) at 3, 14, and 20 days compared to the normal control group, but the differences did not reach statistical significance (*P* > .05) except that at 14 days. Although the effect of eCMC was slightly higher than that of Vaseline gauze, no significant difference was observed (*P* > .05) (Figure 3D).

These findings suggest that eCMC may have a potential influence on the expression of FGF2, highlighting its possible role in promoting tissue repair and regeneration. However, further investigations are necessary to elucidate the clinical significance and underlying mechanisms associated with these observations.

(5) *Effects of eCMC on keratinocyte growth factor*

The keratinocyte growth factor (FGF7), also known as KGF (keratinocyte growth factor), is induced in mesenchymal cells by inflammation or tissue damage and is associated with tissue damage.

The results demonstrated that Vaseline gauze significantly enhanced the expression of keratinocyte growth factor (FGF7) on days 3 and 20 when compared to normal controls (*P* < .05). On the other hand, eCMC did not exhibit any effect on keratinocyte growth factor (FGF7) expression on the 3rd day and 20th day (*P* < .05). However, eCMC showed a slight increase in the expression of keratinocyte growth factor (FGF7) on the 14th day. Nonetheless, these differences did not reach statistical significance when compared to normal tissue (*P* > .05). These findings suggest that eCMC may possess a certain degree of protective function against tissue damage (Figure 3E). Further investigations are required to fully elucidate the potential role of ECMC in modulating keratinocyte growth factor (FGF7) expression and its implications for tissue healing and repair.

(6) *Effects of eCMC on transforming growth factor β1*

The transforming growth factor β1 (TGF-β1) is a secreted protein with a variety of cellular functions, including the control of cell growth, cell proliferation, cell differentiation, and apoptosis. It is closely related to tissue fibrosis and scarring.

The results revealed that Vaseline gauze significantly increased the expression of TGF-β1 on days 3 and 20 in comparison to normal controls. Interestingly, on the 3rd day, eCMC exhibited a certain inhibitory effect on the expression of TGF-β1. However, on the 14th and 20th days, eCMC slightly increased the expression of TGF-β1, although the comparison with normal tissues did not reach statistical significance (*P* > .05). These findings suggest that eCMC may have a certain inhibitory effect on tissue fibrosis and scarring (Figure 3F). Further investigations are required to fully understand the potential impact of eCMC on modulating the expression of TGF-β1 and its implications for fibrosis and scarring in tissue healing and regeneration.

(7) *Effects of eCMC on transforming growth factor β3*

The transforming growth factor β3 (TGF-β3) is a pleiotropic cytokine that can regulate immune function, proliferation, especially epithelial-mesenchymal transition, and plays an important role in the repair of burn skin epithelium.

The results indicated that both eCMC and Vaseline gauze increased the expression of TGF-β3 on day 3 compared to normal controls. However, on the 14th and 20th days, the effect of Vaseline gauze in increasing the expression of TGF-β3 diminished, while eCMC continued to enhance the expression of TGF-β3, showing a gradual increasing trend. Notably, on the 14th day and 20th day, the effect of eCMC on the expression of TGF-β3 was significantly higher than that of Vaseline gauze (*P* < .05). These results suggest that eCMC exhibits a regulatory effect on the epithelial-mesenchymal transition of the skin epithelium, thereby promoting the formation and maturation of new skin (Figure 3G).

### Effects of eCMC on multiple tissue cytokine array at different time points

Furthermore, the effect of eCMC on tissue healing and regeneration following burn injury was evaluated by detecting 43 endogenous cytokines in the tissue using Cytokine Array analysis. This analysis provided insight into the various cytokines present in the tissue and allowed for the assessment of the effects of eCMC on their expression levels.

**Figure 4.**
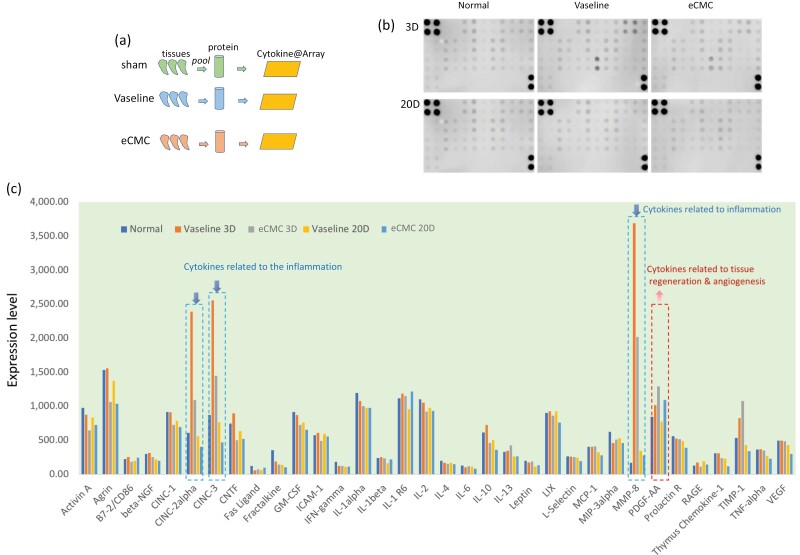
Cytokine Microarray. (A) Experimental Procedure of Cytokine Microarray. (B) Scanned Images of Cytokine Microarray. (C) eCMC Effectively Modulated the Endogenous Cytokines in the Tissue, Resulting in the Inhibition of the Inflammatory Response and Promotion of Tissue Regeneration During the Treatment of Burn Injuries

As mentioned above, tissue protein was extracted from 6 tissue samples in each group, and then each group was mixed together to form a test sample. The original scan of the detection results is shown in Figure 4B. Interestingly, eCMC exhibits a significant ability to inhibit factors associated with tissue inflammation. Particularly noteworthy are CINC-2, MMP8, and CINC3, which are effectively suppressed by eCMC. This antiinflammatory response persists over time, with the strongest effect observed on day 3, surpassing the effect observed on day 20 (Figure 4C).

(1) *3-day comparison of eCMC and vaseline gauze groups*

Three days after the burn injury, the findings revealed that eCMC significantly increased the expression of 8 cytokines, namely, MCP-1, INF-r, Leptin, MIP3, IL6, IL 13, Fas-L, PDGF-AA, and TIMP-1. Notably, the increase in PDGF-AA holds significant biological importance as it signifies the potential of eCMC to promote tissue regeneration. These results suggest that eCMC exhibits a multifaceted effect on various cytokines involved in the healing process, further supporting its efficacy in facilitating tissue regeneration.

**Figure 5.**
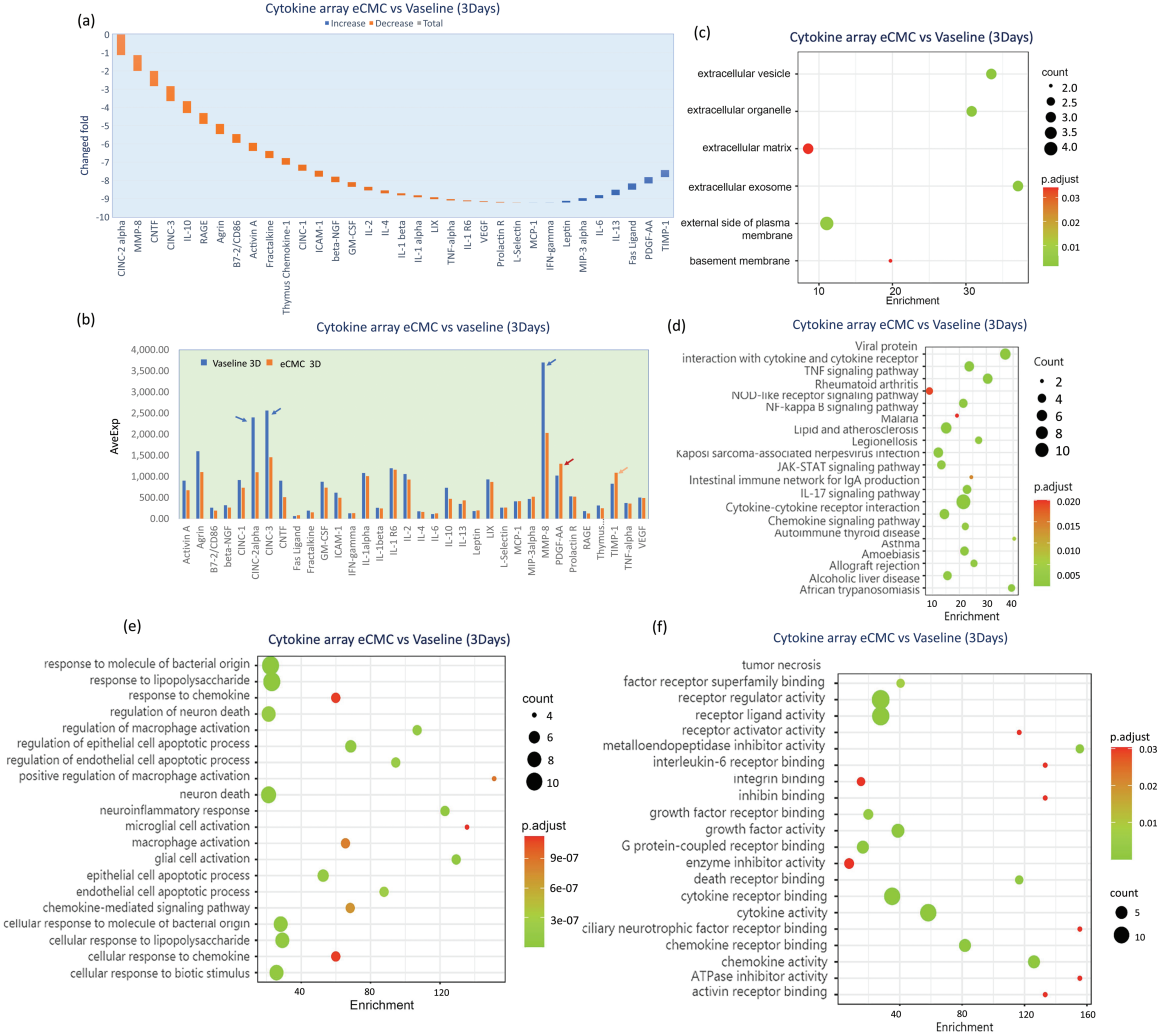
eCMC-Regulated Tissue Endogenous Cytokine (Classification)—3 Days. (A) Changed Fold of Cytokines. (B) Expression Level of Cytokines. (C) Cytokines Related to Extracellular Matrix. (D) Cellular Signaling Pathways Related. (E) Cytokine Related. (F) Cytokine and Receptor Related

Moreover, eCMC demonstrated a significant inhibitory effect on 25 endogenous cytokines. Notably, the most prominent inhibitory effects were observed on CINC-2, MMP8, and CINC3, which are closely associated with tissue inflammation. This finding indicates that eCMC possesses a strong potential to suppress tissue inflammation, leading to improved healing outcomes (Figure 5A and B). Interestingly, eCMC exhibited activation of factors linked to the extracellular matrix, while simultaneously inhibiting ECM degradation (Figure 5C). In addition, eCMC exerted regulatory effects on numerous cellular signaling pathways, particularly enhancing the activity of cytokine receptors involved in interactions with viral proteins (Figure 5D). This result suggests that eCMC may possess antiviral capabilities. Furthermore, through in-depth analysis of these endogenous cytokines, it was observed that eCMC significantly inhibited factors associated with bacterial infection and tissue inflammatory responses, as well as nerve injury and cell death, while activating factors associated with tissue regeneration (Figure 5E). Similarly, the corresponding cytokine receptors exhibited similar responses (Figure 5F).

(2) *20-day comparison results between eCMC and vaseline gauze groups*

Twenty days after the burn injury, the results showed that a total of 7 cytokines were increased by eCMC, including INF-r, Leptin, CD86, IL1, IL-1b, PDGF-AA, and Fas-L. The increase of PDGF-AA was especially obvious, significantly higher than the 3-day increase. This has important biological implications, indicating that eCMC can promote tissue regeneration, and the effect persists.

**Figure 6.**
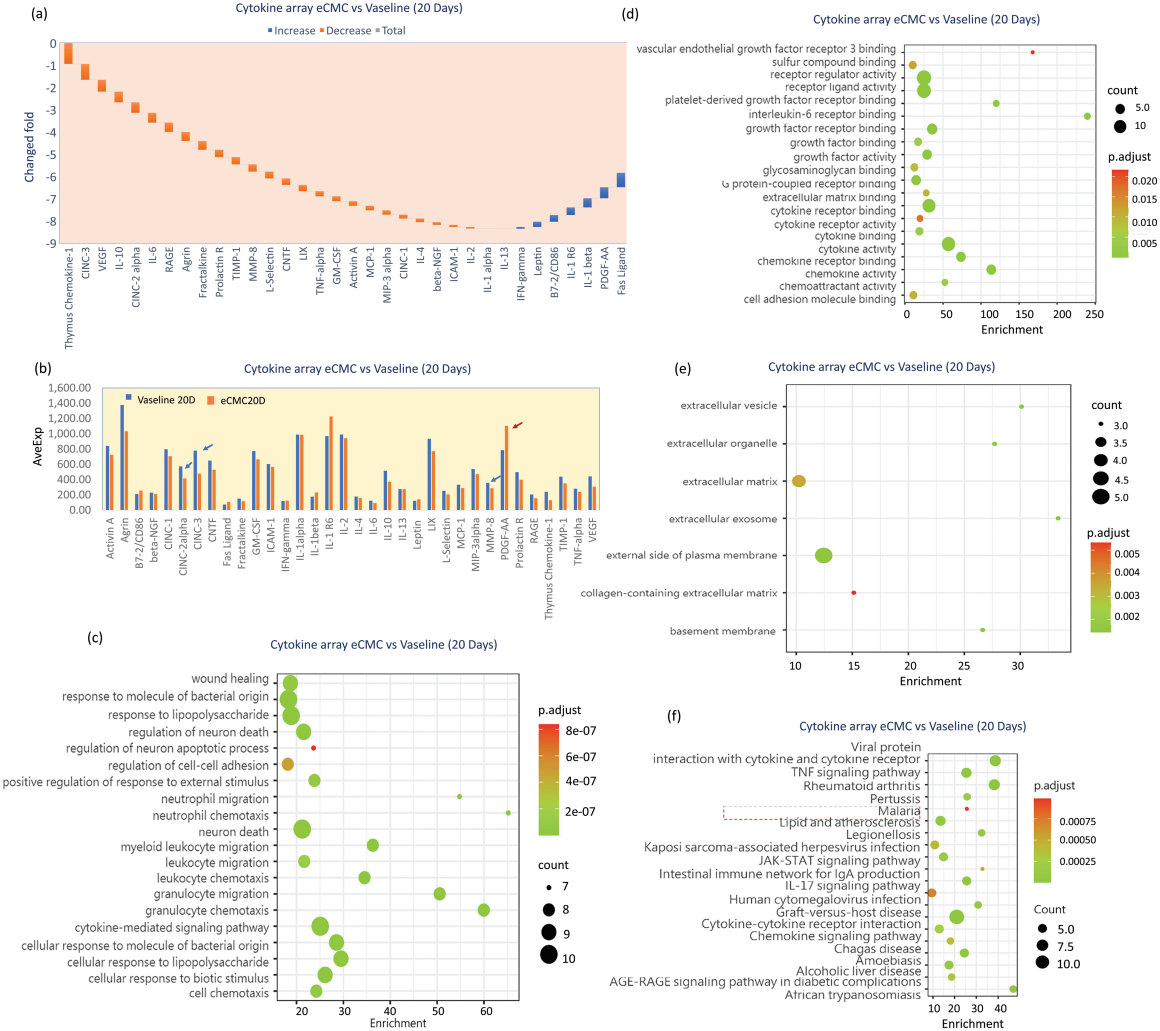
eCMC-Regulated Tissue Endogenous Cytokines and Promotes Tissue Regeneration in Burn Injury—20 Days. (A) Changed Fold of Cytokines. (B) Expression Level of Cytokines. (C) Cytokine Related. (D) Cytokine and Receptor Related. (E) Cytokines Related to Extracellular Matrix. (F) Cellular Signaling Pathways Related

In addition, eCMC significantly inhibited 27 endogenous cytokines, the most important of which were CINC-2, MMP8, and CINC3, which were all related to tissue inflammation, indicating again that eCMC can significantly inhibit tissue inflammation and that this function persists over time (Figure 6A and B). Also, through the in-depth analysis of these endogenous cytokines, it was found that the factors associated with tissue damage, bacterial infection, and tissue inflammatory responses, leukocyte infiltration were significantly inhibited, while leukocyte chemotactic factors were activated. Of particular note is the inhibition of intercellular adhesion signaling molecules, which are important for tissue regeneration and shaping (Figure 6C). The associated cytokine receptors also responded similarly, notably the activation of VEGF receptor 3 by eCMC, suggesting that eCMC can promote angiogenesis (Figure 6D). Notably, some factors associated with extracellular matrix were activated at 20 days, while ECM degradation was inhibited, and collagen-related extracellular matrix activity was reduced, suggesting that eCMC regulates tissue shaping (Figure 6E). Moreover, many signaling pathways were regulated and similar to 3 days, especially the activity of cytokine receptors interacting with viral proteins was enhanced and persisted (Figure 6F). This result suggests that eCMC has potential antiviral ability.

## DISCUSSIONS

Numerous studies have highlighted the importance of wound hemostatic products that not only effectively stop bleeding but also promote wound healing, while demonstrating tissue compatibility and absorbability.^12,13^ Our previous research has demonstrated that eCMC possesses all of these desirable qualities. When eCMC forms a gel, such as in the case of eCMC, it acts as a scaffold, providing a supportive environment for the patient’s native cells to reside, flourish, and regenerate tissue. The open scaffold, without cross-linking, allows for cellular infiltration and the growth of new capillaries. Furthermore, the release of VEGF and PDGF from host cells, stimulated by eCMC, aids in the formation of well-vascularized new skin tissues.^6^ The application of eCMC is targeted and local, being applied externally to the site of a burn injury following the removal of debris. As such, the procoagulant effects of eCMC are primarily external and localized rather than systemic. Consequently, eCMC does not influence the systemic coagulation system, avoiding the potential for widespread coagulation disturbances.

The gel-forming properties of eCMC create an ideal microenvironment for modulating endogenous cytokines. Through its interaction with cellular receptors on cell membranes, eCMC activates cellular signaling and modulates the expression of key cytokines, including VEGF, PDGF, EGF, FGF2, FGF7, TGF-β1, and TGF-β3, among others.^14^ Notably, VEGF and PDGF, stimulated by eCMC, play crucial roles in promoting angiogenesis, the growth of new blood vessels.^15,16^ At the same time, eCMC stimulates the production of FGF7 and other cytokines, which contribute to tissue protection and reduce apoptosis. In addition, the stimulation of TGF-β3 and other cytokines by eECMC facilitates cellular transformation and infiltration, leading to enhanced skin regeneration and reepithelialization. These effects collectively promote wound healing and tissue regeneration. eCMC demonstrated significantly increase in growth factors related to tissue regeneration and repair, such as PDGF-AA, thereby promoting tissue regeneration, and this effect was persistent over time as it was higher at 20 days as compared to 3 days. The eCMC modulated endogenous cytokines, whereas the gel provided the perfect microenvironment for it molecules to bind to the cellular receptors on cell membranes to activate cellular signaling for modulating endogenous cytokines, such PDGF-AA et al.^17^ These current research results perfectly explain the molecular biological mechanism of eCMC in promoting wound healing in preclinical and clinical applications.

In actual clinical practice, wound infection is of critical importance as it relates to burn debridement, grafting, and healing of the donor sites of critically injured burn patients. Therefore, as has been demonstrated in past studies, some various antiinflammatory products, such as silver nitrate, were added to the wound site, to attempt to block these harmful cytokines. While these substances inhibit bacteria, they often are toxic to tissue cells, inhibiting tissue regeneration and healing. In the current study, we got an unexpected result, revealed that the novel hemostatic, eCMC not only facilitates hemorrhage control, but it also decreased inflammatory cytokines, including MMP8, CINC1, and CINC3,^18,19^ allowing for a reduction the infection and inflammation risk, thus allowing for a wound environment conducive to wound healing. This finding further illustrates those patients the effect of the product as it relates to wounds treated with eCMC, as it could demonstrate the ability to have prevent fewer wound infections in the in vitro model.

Due to the limitation of small animal model, more studies in vivo of newly designed large animal models will be conducted to evaluate eCMC treatment in partial-thickness wound healing.

## CONCLUSIONS

The eCMC emerges as an ideal solution for wound hemostasis and healing, offering a multifaceted approach to promote tissue regeneration. By inducing a range of endogenous cytokines, with particular emphasis on VEGF and PDGF, eCMC plays a vital role in accelerating tissue healing. Simultaneously, it effectively inhibits inflammatory cytokines like CINC-1, CINC-2, and MMP-8, thereby reducing the risk of wound infection and facilitating the healing process. Despite many studies which promote the use of anticoagulants to facilitate recovery of the zone of stasis, this product, despite being procoagulant in nature, facilitates healing through its affect on multiple cytokines. Despite the products procoagulant effect it does not adversely inhibit healing, but rather promotes such healing, due to the up regulation of PDGF and VEGF, as demonstrated in the cytokine array, thus demonstrating the very complex and multifactorial nature of healing of the burn wound that is affected by this product. Regardless of the findings of this study, the precise mechanism underlying eCMC’s regulation of these cytokines is unknown and warrants further investigation. Future studies employing in vitro, and in vivo models are essential to unveil the true potential of eCMC in wound healing.

## SUPPLEMENTARY MATERIAL

Supplementary material is available at *Journal of Burn Care & Research* online.

irae124_suppl_Supplementary_Figure_S1

## References

[1] Ramli NA , Wong TW. Sodium carboxymethylcellulose scaffolds and their physicochemical effects on partial thickness wound healing. Int J Pharm. 2011;403(1–2):73–82. https://doi.org/10.1016/j.ijpharm.2010.10.023 20974238

[2] Rodrigues C , de Assis AM, Moura DJ, et al New therapy of skin repair combining adipose-derived mesenchymal stem cells with sodium carboxymethylcellulose scaffold in a pre-clinical rat model. PLoS One. 2014;9(5):e96241. https://doi.org/10.1371/journal.pone.0096241 24788779 PMC4008527

[3] Gaihre B , Jayasuriya AC. Fabrication and characterization of carboxymethyl cellulose novel microparticles for bone tissue engineering. Mater Sci Eng C Mater Biol Appl. 2016;69(C):733–743. https://doi.org/10.1016/j.msec.2016.07.060 27612767 PMC5019123

[4] Ferretti L , Qiu X, Villalta J, Lin G. Efficacy of BloodSTOP iX, surgicel, and gelfoam in rat models of active bleeding from partial nephrectomy and aortic needle injury. Urology. 2012;80(5):1161.e1–1161.e6. https://doi.org/10.1016/j.urology.2012.06.048 22921708

[5] Li H , Wang L, Alwaal A, et al Comparison of topical hemostatic agents in a swine model of extremity arterial hemorrhage: BloodSTOP iX Battle Matrix vs. QuikClot combat gauze. Int J Mol Sci. 2016;17(4):545. https://doi.org/10.3390/ijms17040545 27077848 PMC4849001

[6] Ju S , Wang K, Qiao L, et al Application of BloodSTOP iX wound heal nanocellulose matrix for burn wound care. J Surg Res. 2021;04(1):14–31.

[7] Liang M , Chen Z, Wang F, Liu L, Wei R, Zhang M. Preparation of self-regulating/anti-adhesive hydrogels and their ability to promote healing in burn wounds. J Biomed Mater Res B Appl Biomater. 2019;107(5):1471–1482. https://doi.org/10.1002/jbm.b.34239 30296361

[8] Calienno R , Curcio C, Lanzini M, Nubile M, Mastropasqua L. In vivo and ex vivo evaluation of cell-cell interactions, adhesion and migration in ocular surface of patients undergone excimer laser refractive surgery after topical therapy with different lubricant eyedrops. Int Ophthalmol. 2018;38(4):1591–1599. https://doi.org/10.1007/s10792-017-0627-y 28676989

[9] Garrett Q , Simmons PA, Xu S, et al Carboxymethylcellulose binds to human corneal epithelial cells and is a modulator of corneal epithelial wound healing. Invest Ophthalmol Vis Sci. 2007;48(4):1559–1567. https://doi.org/10.1167/iovs.06-0848 17389485

[10] Peng PD BR-MB , Banie BL, Wang WG, Lin LG, Lue FT. Carboxymethylcellulose activates dermal cells and adipose-derived stem cells through Wnt/ß-catenin pathway. J Surg Res. 2021;04(01):128–141.

[11] Cai EZ , Ang CH, Raju A, et al Creation of consistent burn wounds: a rat model. Arch Plast Surg. 2014;41(4):317–324. https://doi.org/10.5999/aps.2014.41.4.317 25075351 PMC4113688

[12] Peng HT. Hemostatic agents for prehospital hemorrhage control: a narrative review. Mil Med Res. 2020;7(1):13. https://doi.org/10.1186/s40779-020-00241-z 32209132 PMC7093954

[13] Amit M , Binenbaum Y, Cohen JT, Gil Z. Effectiveness of an oxidized cellulose patch hemostatic agent in thyroid surgery: a prospective, randomized, controlled study. J Am Coll Surg. 2013;217(2):221–225. https://doi.org/10.1016/j.jamcollsurg.2013.03.022 23870217

[14] Douglas HE. TGF-ss in wound healing: a review. J Wound Care. 2010;19(9):403–406. https://doi.org/10.12968/jowc.2010.19.9.78235 20852569

[15] Zhang L , Hu C, Xu W, Wu D, Lei S. Advances in wound repair and regeneration: systematic comparison of cell free fat extract and platelet rich plasma. Front Chem. 2022;10(1):1089277. https://doi.org/10.3389/fchem.2022.1089277 36618858 PMC9811168

[16] Kim E , Ham S, Jung BK, Park JW, Kim J, Lee JH. Effect of baicalin on wound healing in a mouse model of pressure ulcers. Int J Mol Sci. 2022;24(1):329. https://doi.org/10.3390/ijms24010329 36613772 PMC9820804

[17] Wang L , Yang T, Ding L, Ye X, Wu L. Platelet-derived growth factor AA-modified electrospun fibers promote tendon healing. J Biomater Appl. 2022;37(6):1018–1028. https://doi.org/10.1177/08853282221139274 36411499

[18] Annamalai P , Thangam EB. Local and systemic profiles of inflammatory cytokines in carrageenan-induced paw inflammation in rats. Immunol Invest. 2017;46(3):274–283. https://doi.org/10.1080/08820139.2016.1248562 27967265

[19] Tang SE , Liao WI, Pao HP, et al Poloxamer 188 attenuates ischemia-reperfusion-induced lung injury by maintaining cell membrane integrity and inhibiting multiple signaling pathways. Front Pharmacol. 2021;12(7):650573. https://doi.org/10.3389/fphar.2021.650573 34335242 PMC8319770

