# Supplementary material for: Regulating Tissue Growth Factors for Healing With Etherified Carboxymethylcellulose Matrix
Source: J Burn Care Res. 2024 Jul 2;45(6):1566–76. doi: 10.1093/jbcr/irae124 (PMC11565206; doi:10.1093/jbcr/irae124)
Supplement: irae124_suppl_Supplementary_Figure_S1 [file irae124_suppl_supplementary_figure_s1.pptx]

## Slide 1
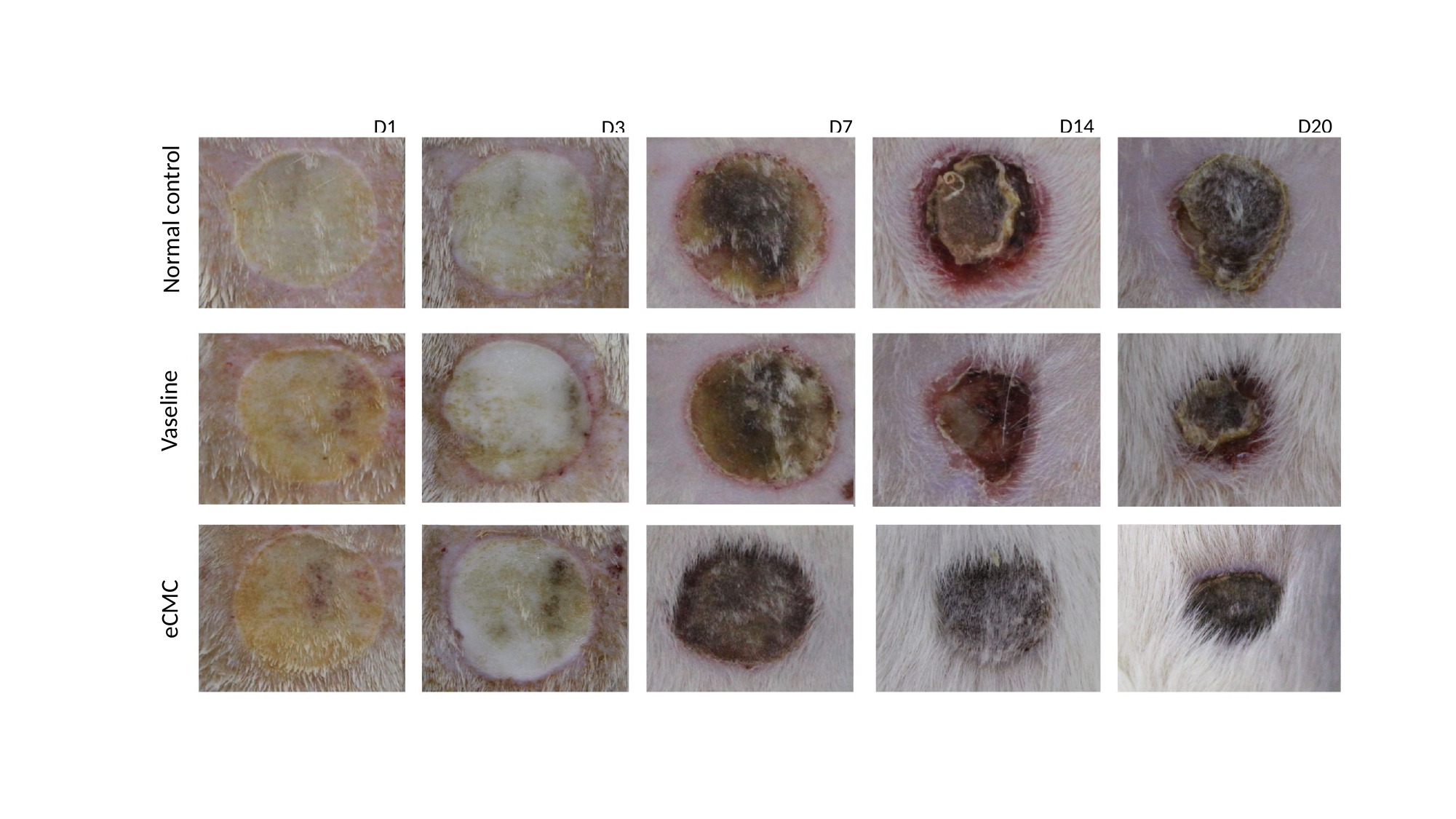

D14
D20
D7
D1
D3
Normal control
Vaseline
eCMC

## Slide 2
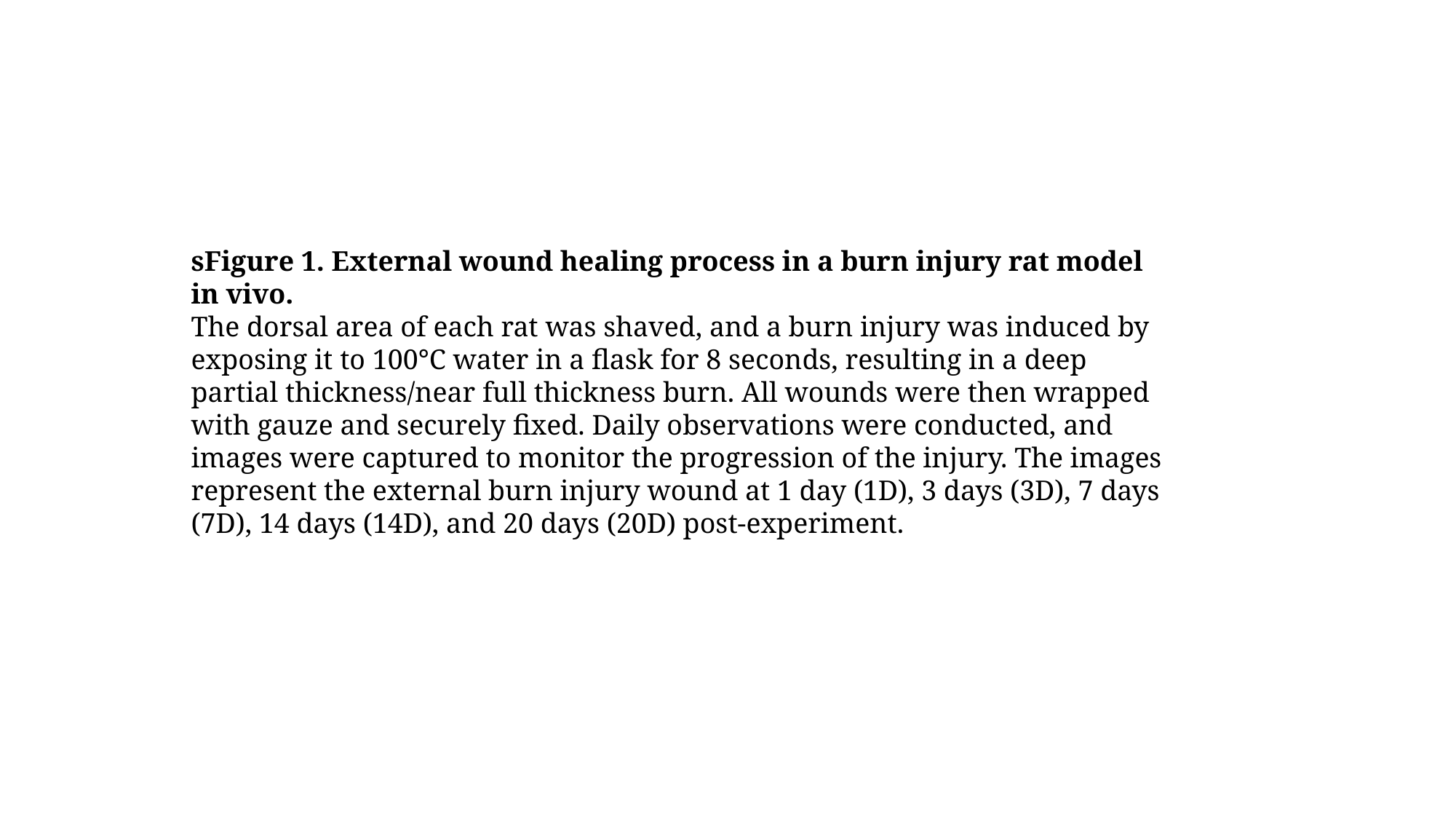

sFigure 1. External wound healing process in a burn injury rat model in vivo.
The dorsal area of each rat was shaved, and a burn injury was induced by exposing it to 100°C water in a flask for 8 seconds, resulting in a deep partial thickness/near full thickness burn. All wounds were then wrapped with gauze and securely fixed. Daily observations were conducted, and images were captured to monitor the progression of the injury. The images represent the external burn injury wound at 1 day (1D), 3 days (3D), 7 days (7D), 14 days (14D), and 20 days (20D) post-experiment.
